# Trends in Protein-Based Biosensor Assemblies for Drug Screening and Pharmaceutical Kinetic Studies

**DOI:** 10.3390/molecules190812461

**Published:** 2014-08-18

**Authors:** Ana M. Gonçalves, Augusto Q. Pedro, Fátima M. Santos, Luís M. Martins, Cláudio J. Maia, João A. Queiroz, Luís A. Passarinha

**Affiliations:** CICS-UBI Centro de Investigação em Ciências da Saúde, Universidade da Beira Interior, 6201-506 Covilhã, Portugal; E-Mails: ggmargarida@gmail.com (A.M.G.); apedro@fcsaude.ubi.pt (A.Q.P.); ftxsantos@gmail.com (F.M.S.); lmcmartins1@hotmail.com (L.M.M.); cmaia@fcsaude.ubi.pt (C.J.M.); jqueiroz@ubi.pt (J.A.Q.)

**Keywords:** biosensor, pharmacokinetics, enzymes, antibodies, plasma proteins, nano-composites

## Abstract

The selection of natural and chemical compounds for potential applications in new pharmaceutical formulations constitutes a time-consuming procedure in drug screening. To overcome this issue, new devices called biosensors, have already demonstrated their versatility and capacity for routine clinical diagnosis. Designed to perform analytical analysis for the detection of a particular analyte, biosensors based on the coupling of proteins to amperometric and optical devices have shown the appropriate selectivity, sensibility and accuracy. During the last years, the exponential demand for pharmacokinetic studies in the early phases of drug development, along with the need of lower molecular weight detection, have led to new biosensor structure materials with innovative immobilization strategies. The result has been the development of smaller, more reproducible biosensors with lower detection limits, and with a drastic reduction in the required sample volumes. Therefore in order to describe the main achievements in biosensor fields, the present review has the main aim of summarizing the essential strategies used to generate these specific devices, that can provide, under physiological conditions, a credible molecule profile and assess specific pharmacokinetic parameters.

## 1. Introduction

The biopharmaceutical industry invests a great amount of time and resources in drug discovery and development. Devising a process effective in terms of time and cost and that covers the target identification and validation of new therapeutic targets, as well as the research of hit and lead molecules that could be used as therapeutic agents in clinical treatments, is currently a challenge for the drug discovery industry [1,2].

The growing number of either synthetic or natural new pharmacological compounds, alongside with the knowledge resulting from genomics, which has led to an increasing number of pharmaceutical targets without known molecular modulators, has triggered the need for new screening models capable of reducing the attrition rates during drug development [1]. Over the past two decades, several screening methodology approaches were used, including fragment screening [3], structure-based design [4], virtual screening [5] and high-throughput screening (HTS) [2].

**Figure 1:**
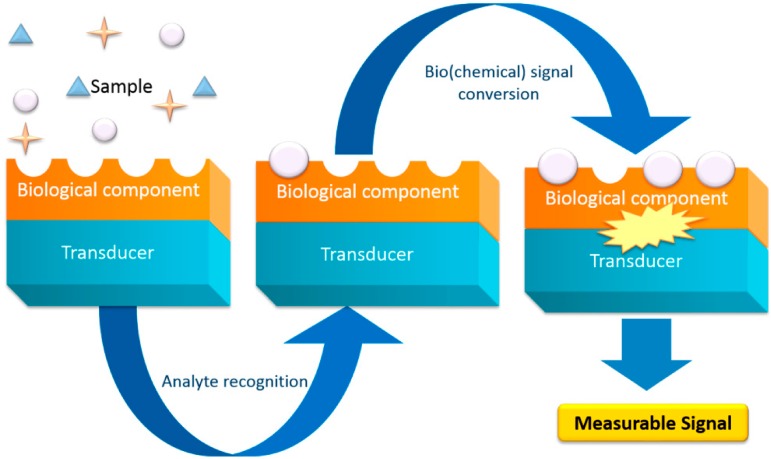
Operative scheme of a standard biosensor. The biological part is either integrated or closely associated with the physical transducer, and behave as a recognition element, capable to detect a specific biological analyte. Once the interaction takes place, the bio(chemical) signal generated will be converted by a physical transducer in a measurable discrete or continuous signal, whose intensity, could be directly or inversely proportional to the analyte concentration.

Presently, most of the screening techniques used to report the binding of the ligand to its receptor are based on the measurement of fluorescence (e.g., fluorescence anisotropy [6,7], time resolved energy transfer [8], fluorescence correlation spectroscopy [9], fluorescence intensity distribution analysis [10]) or scintillation (e.g., scintillation proximity assay [11]). These approaches offer high sensitivity with rapid determination of affinity, efficacy and kinetics of drug-receptor interaction [12]. However, the required labeling steps represent extra time and cost demands for the process and in some cases could interfere with the molecular interaction by the blocking of the binding site, leading to false negatives. On the other hand, fluorescent compounds are invariably hydrophobic, and in many screens, background binding is a significant problem, leading to false positives [13], so the development of platforms with sufficient throughput to be applied in drug discovery and high sensitivity in order to provide detailed information about the molecular efficacy and interactions without labeling is essential. Also, it is important that such technology be able to determine pharmacokinetic parameters, namely in the process of adsorption, distribution, metabolism, excretion and toxicity (ADMET) [1]. Over the years, increasing efforts had been made to improve the understanding of molecular features that lead to a successful identification of the potential drug liabilities by the prediction of human pharmacokinetics. Biosensors are devices composed by two main parts: a biological derived sensing element (like enzymes, antibodies, cells and nucleic acids) and a physical transducer (e.g., electrochemical, piezoelectric, calorimetric and optical), capable of detecting specific analytes in a real-time mode, and converting their presence or concentration into an electric readout signal (Figure 1) [14]. Biosensors, in particular the electrochemical and optical biosensors that constitute the focus of this review, are advantageous tools for the drug development industry, and can be found both in the screening process and in the pharmacokinetics evaluation. Although electrochemical and optical biosensors can be used in both applications, is also true that electrochemical sensors are predominantly used to detect the drugs and assess their toxicity, whereas optical sensors are used specifically to predict a drug’s pharmacokinetic profile. This review intends to illustrate the importance of protein- based biosensors, their accomplishments and future perspectives.

## 2. Proteins as Biolog ical Recognition Elements

The selection of the biological recognition elements is a crucial step in a construction of a biosensor [15]. The selectivity and specificity of the biosensor depends on the correct selection of the biological component that must only interact with the analyte of interest but not with other substances present in the target sample [15,16,17]. Then, the biological element should be capable to detect the presence, activity, and quantify a specific analyte in solution [18]. Therefore, is fundamental that the assembly process assure the stability and sensitivity of the recognition element. Proteins and enzymes are unstable structures that must to be immobilized in order to be used as biosensors. Since the reuse of the enzyme is only possible if the activity is retained for several cycles, enzyme stabilization is a crucial step [19].

Stabilization has been attributed to a rigid conformation of the immobilized biocatalyst, which prevents unfolding of enzyme and inactivation of its active site. So, along with stability, immobilization also enables the efficient recovery of the enzyme from the reaction environment as well as their use in continuous operation [20]. However, a specific immobilization process will not always lead to enzyme stabilization.

There are three major routes for performing immobilization: the binding to a support [21,22,23], encapsulation or entrapment [24] and cross-linking (carrier free) [25]. The main drawbacks and advantages of each technique are depicted in Table 1. The immobilization of the enzymes on a support may alter its performance in different processes such as: selective hydrolysis or oxidation, kinetic resolution of racemic mixtures or influence the kinetic controlled synthesis [26] In fact, random immobilization on a wrong support may produce even a decrease of enzyme stability [19]. For that, more demanding technologies like tailor-made heterofunctional supports and site-directed mutagenesis are employed. Since they allow control over enzyme immobilization, the orientation of the protein on the support surface, and the intensity of enzyme interaction with the support, these techniques have led to unprecedented target protein stabilization [20,27]. Furthermore, these new methods can improve the activity and even the selectivity of the immobilized protein through directed rigidification of selected areas of the protein [28].

**Table 1:** Summary of the main advantages and drawbacks of typical immobilization methods applied in pharmaceutical fields.

Method/Binding Nature	Advantages	Drawbacks
*Adsorption*	Simple and easy; Limited loss of protein activity.	Desorption; Random orientation; Non-specific adsorption.
*Covalent-coupling*	Stable; No diffusion barriers; Short response time.	Coupling with toxic product; Random orientation; Poor reproducibility.
*Cross-linking*	Simple technique.	High protein activity loss.
*Affinity Interaction*	Perfect control of enzyme orientation during immobilization procedure.	Require the presence of specific groups on the enzyme.
*Entrapment*	No chemical reaction between the monomer and the enzyme that could affect the activity; Several types of enzymes can be immobilized within the same polymer.	Enzyme leakage; Diffusion Barrier; Require high concentration of monomer and enzyme in the electropolymerization step.

The selection of an appropriate carrier material, in order to prepare an effective immobilization procedure, is also relevant. Nanomaterials, as carriers or entrapment agents are gaining a prominent place within immobilization methods, as they offer the possibility of tuning their pore diameters, hardness, hydrophobicity/hydrophilicity ratio as well as the control of the magnetic properties and conductivity, and thus increase the precision of enzyme immobilization control [20,29]. Nanomaterials and nanostructures also generally provide a large surface area and low mass-transfer resistance, which enables better interaction with the enzyme, increases immobilization efficiency, and enhances the long-term storage and recycling stability of the enzyme [29].

In summary, all the previously referred techniques have the same aim, the production of a stable layer with an attached biomolecule with sufficient binding sites, capable of maintaining its structure and activity to recognizing the target analyte. On the other hand, factors such as accuracy, sensor-to-sensor reproducibility and operation lifetime are factors influenced by the stability of the binding between the biological recognition element and the transducer. Therefore, the ideal method for a suitable immobilization should be a compromise between sensitivity and stability [17,18]. So far, biological recognition elements such as enzymes, antibodies, DNA, receptors, organelles, microorganisms, and some plant and animal tissues have allowed the detection of a wide range of biological or chemical compounds [30]. In particular, protein-based biosensors have gained applicability in several fields such as health care, food safety, environmental monitoring, drug screening, and pharmaceutical studies [31].

### 2.1. Enzymes

The first proposed biosensors were an enzyme-based device developed by Clark and Lyons in 1962 to measure glucose levels. Since then, enzyme-based biosensors have seen a massive growth in terms of applications [14]. As biological molecules responsible for thousands of metabolic processes, enzymes are the oldest and still the most used recognition element in biosensors. Due to their special structural active site arrangement, enzymes have an optimum specificity for a substrate molecule, having the ability to detect target compounds in a complex mixture [14]. In some cases, enzymes incorporate small non-protein chemical groups (e.g., cofactors) into the active site structures, which can also help in determination of substrate specificity. Based on catalytic action and binding capabilities for specific detection, enzymes provide to the sensor the ability to detect much lower limits when compared with other binding techniques [15,16]. However, enzymes are often instable which results in a decrease of enzyme activity.

There are several applications of enzyme-based biosensors as a screening methodology in the pharmaceutical industry as shown by Table 2, including the search for new antibiotics and cancer therapeutic drugs, among others. An example of the spread and evolution of enzyme-based biosensors is the penicillin biosensor [32]. Belonging to the most familiar group of β-lactam antibiotics, penicillin, has been used extensively in human medicine to prevent infections caused by bacteria and fungi [33], so the determination of different kinds of penicillin and derivatives, is very important in medicine and pharmaceutical production (e.g., the drug control analysis of antibiotic tablets, capsules and injectables) and this requires a rapid, inexpensive method capable of detecting small amounts of penicillin [34]. Based on this idea, Poghossian and co-workers developed a pH-sensing biosensor with immobilized penicillinase on its active surface. Penicillinase catalyzes the hydrolysis of penicillin to penicillinic acids, which results in a variation of H^+^ ion concentration, leading to a pH change. The result was a biosensor sensitive to penicillin G with a low detection limit of 0.5 µM. Due to the introduction of a diffusion barrier, as shown by the group, in a study of penicillin G, ampicillin, and amoxicillin, the detection limit could be decreased from 0.1 mM for penicillin G to a lower value of 0.5 µg of penicillin per analyzed sample [34,35]. More recently, with a layer-by-layer film containing single graphene nanosheets, preadsorbed with hematein, ionic liquids and penicillinase, Wu and co-workers achieved a low detection limit penicillin biosensor that exhibited excellent performance for penicillin in a linear range from 1.25 × 10^−13^ M to 7.5 × 10^−3^ M and a incredible detection limit of 10^−13^ M [36]. As we can see, when the drugs contain hydrolysable groups, or when there is H^+^ liberation, selective electrodes or potentiometric biosensors, which we will discuss in more detail in this review, are the choice. When oxidized drugs are the focus of the study, amperometric tranducers and oxidases enzymes such as peroxidases, laccase, tyrosinase, and superoxide dismutase are the preferred choice, as shown by Table 2.

**Table 2:** Biosensor performance based on different enzyme immobilization strategies for pharmaceutical applications.

	Immobilized Technique	Analyte	Immobilized Enzyme	Detection Method	Detection Limit	Linear Range	Sensitivity	References
***Adsorption***	Physical Adsorption	H_2_O_2_	HRP	Amperometric	2 μmol∙L^−1^	8.0 μmol∙L^−1^ to 3.0 mmol∙L^−1^	-	[37]
	Layer-by-layer	Glucose	GOD	Amperometric	0.20 mmol∙L^−1^	-	16 µA mmol∙L^−1^∙cm^−2^	[38]
	Layer-by-layer	Glucose	GOD	Amperometric	83 µmol/L	0.5 to 5.5 mmol/L	-	[39]
	Electrochemical doping	Choline	ChOD	Amperometric	-	1 × 10^−7^ to 1 × 10^−4^ M	-	[40]
	Lipidic microenvironement	Lactase	Lactase/GalOD	Amperometric	-	5.6 × 10^−2^ to 3.3 × 10^−1^	-	[41]
***Covalent coupling***		Lactate	LDH	Amperometric	1 μM	5 to 90 μM	0.0106 μA/μM	[42]
		Dopamine	Tyrosinase	Amperometric	-	5–120 μM	-	[43]
		Dopamine	Tyrosinase	Amperometric	-	1–200 µM	232.5 mA∙M^−1^∙cm^−2^	[44]
		Urea	Urease	Amperometric	-	0.16–5.02 μM	-	[45]
		Urea	Urease	Amperometric	0.02 mM	0.1 to 0.7 mM	4.5 µA/mM	[46]
***Cross-linking***		Glucose	GOD	Amperometric	1 µM	5 × 10^−5^ to 1.2 × 10^−2^ M	21.7 A/mM∙cm^2^	[47]
		Glucose	GOD	Amperometric		70–420 mg∙dL^−1^	-	[48]
		MSG	(l-GLOD)/(l-GLDH)	Amperometric	0.02 mg/L	0.02 to 1.2 mg/L	-	[49]
***Affinity***	Biotin/avidin	Urea	Urease	ChemFEC	-	10^−4^ to 10^−1^ M	-	[23]
	Chelation	Paraoxon	AChE	Electrochemical	10−12 M	10^−8^ to 10^−13^	-	[50]
***Entrapment***	Electropolymerization	Glucose	GOD/HRP	Amperometry	3 × 10^−5^ M	(3.00 × 10^−5^ to 2.43 × 10^−3^ M	7.01 ± 0.18 μA∙mM^−1^∙cm^−2^	[51]
	Photopolymerization	Dichlorvos	AChE	Amperometry	9.6 × 10^−11^ mol∙L^−1^	2 × 10^−10^–1 × 10^−8^	-	[52]
	Silica-sol-gel	Glucose	GOD	Amperometry	50 μM	0.2–20 mM	196 nA/mM	[53]
	Polysaccharide-based gel	Ethanol	ADH	Amperometry	0.52 μM	-	0.1646 AM^−1^·cm^−2^	[54]
	Carbon Paste electrodes	Dopamine	HRP	Amperometry	9.0 × 10^−6^ mol∙L^−1^	9.9 × 10^−5^ to 1.6 × 10^−3^ mol∙L^−1^	-	[55]
	Agarose	Dopamine	Tyrosinase	Amperometry	9.0 × 10^−7^	2.0 × 10^−6^ to 1.0 × 10^−5^	-	[56]
	Sol-gel	Xantine	XO, SOD and HRP	Fluorescence	20 nM	0–3.5 µM	-	[57]

Enzyme-based biosensors could also be found in the search for inhibitors [16]. An example is the work performed by Nordin and co-workers. Based on SPR technology and several kinases (serine, threonine and tyrosine), information on the relationship between inhibitor structure and function was collected [58]. More recently the special catalytic features of the enzymes could be incorporated in nucleic acid biosensors to report and detect DNA [59,60].

### 2.2. Antibodies

Antibodies (Abs) or immunoglobins (Igs) are “Y” shaped glycoproteins capable of recognizing a specific antigen. The amino acid sequence in the variable region confers the specificity of the Ab to the Ag, and so the established binding is highly specific and selective [61]. For all these features, Abs were seen as a good recognition molecule to be used in the construction of a biosensor [62].

Despite the fact that the application of immunological compounds as sensing agents was described by Yalow and Berson in the 50s [63], it was not until 1987 that Vo-Dinh and co-workers described the detection of a chemical carcinogen using an optic-based immunosensor [16]. Based on antigen-antibody (Ag-Ab) interactions, where an Ag or an Ab are immobilized on a solid surface [18,63], this device could be coupled to a wide variety of transducers to measure the signal resulting from Ag-Ab interactions [16,18]. However, the two transduction methods more frequently used in immunosensors are the optical and electrochemical ones. The detection can be direct or indirect, meaning that detection of Ag-Ab interactions is in real time or it requires a label compound such as an enzyme or a fluorescent molecule and a secondary reaction to produce the signal [18,63].

The high affinity of Ag-Ab recognition offers a high specificity to the immunosensor [16]. In particular, Abs are the critical part in the immunosensor assembly, defining its sensitivity, stability and specificity [16,64], so an optimized Ab immobilization procedure allows its orientation in the sensor, crucial for maintain its stability and allowing the interaction with the analyte [17]. Nevertheless, an adequate sensitivity and reproducibility is only obtained by applying high affinity Abs [18,64]. Moreover, the Ag-Ab interaction may be irreversible, which leads to harsh conditions for biosensor regeneration [18,64]. Despite this, recombinant Ab and Ab fragments present a viable means to overcome some of these drawbacks, improving the immobilization and, as a result, the selectivity and stability of immunobiosensors [16].

### 2.3. Other Proteins

Plasma proteins such as human serum albumin (HAS) and α1-acid glycoprotein (AGP), are a class of proteins that greatly influence drug distribution. Since the earliest possible ADME data is essential for the selection of lead compounds, and as the overwhelming majority of new drugs are intended to be administrated orally, an assessment of drug bioavailability is imperative. For instance, high affinity for plasma proteins could reduce the free concentration of the drug in the blood stream. Therefore, depending on the drug and the target, a high affinity for plasma proteins may consequently be an advantage or a drawback for efficacy [65]. Therefore, pharmacokinetic evaluation of new drugs through the application of plasma proteins as a biological recognition element and SPR methodology were implemented [66,67].

HAS, the most abundant protein in blood plasma, serves as a transport protein for numerous compounds, especially hydrophobic compounds. The binding of lyophilic drugs to this protein results, in some cases, in higher solubility’s and lowers levels of toxicity [65]. In the study performed by Frostell-Karlsson and co-workers they determined through a SPR technology the binding affinity of HAS to a poll of 19 different drugs; this technology revealed a good correlation with the reported binding HAS levels and required a low samples volume [66].

As illnesses of the century, cancer and HIV are subject to intense investigation in order to develop suitable anticancer chemotherapeutic agents and anti-HIV drugs. For these studies, information about absorption or distribution kinetics is required because the data in the literature regarding this set of molecules suffers from discrepancies and great variability between assays, which is in part due to the complex pharmacokinetics and pharmacodynamics of these classes of molecules [65,68]. To overcome these, Cimitan and co-workers proposed a biosensor based on SPR technology and two human plasma proteins (HAS, AGP). In this study the knowledge of the drug bound percentage and active drug concentration lead to a better understanding of the drug-receptor interaction mechanisms.

## 3. The New Era in Biosensors Nanostructures Assembly

The immobilization process is crucial and could affect the performance of the electron-transfer phenomenon, so much attention has been given in the development of new immobilization procedures, through the combination of techniques and new materials, in order to obtain a high electron transport efficiency between the redox centers of the proteins, and the electrodes, without disrupting the protein native structure [69]. Due to their small size, large surface-to-volume ratios and surface activities, nanodimensional materials, such as nanoparticles, nanotubes, nanofibers and nanorods, represent a promising approach for the construction of biosensing devices [70]. Their unique properties provide a suitable environment for protein immobilization, while maintaining their bioactivity, with a great enhancement in the analytical performance in terms of detection limits and kinetic profile.

In general, noble metal nanomaterials (NMNs) display physical and chemical properties like large surface area, good surface chemistry properties, and high conductivity, ideal to perform redox reactions [71]. These characteristics allow a considerable improvement of the analytical performance, leading to lowered detection limits and reduction of deposition time in comparison to conventional electrodes. The study developed by Yang and co-workers is a clearly example of the previous statement. Through the combination of a platinum nanoparticle-doped sol-gel solution with multi-walled carbon nanotubes (CNT) they created a glucose biosensor with low-potential detection of hydrogen peroxide (range from 1 to 25 mM), a response time lower than 15 s and a remarkable sensitivity (0.98 μA∙mM^−1^∙cm^−2^) when compared to the CNT-based biosensor (0.27 μA∙mM^−1^∙cm^−2^ at 0.1 V) [72]. Another example of this fact is a glucose biosensor proposed by Zhu and co-workers, where glucose oxidase (GOD) and HRP were immobilized. This co-immobilization in an electropolymerized pyrrole (PPy) film on a single wall carbon nanotubes (SWNT)-coated electrode, producing a bienzymatic sensor with lower operational potential (−0.1 V) that led to the preservation of the sensitivity (430 ± 13.4 µAM^−1^∙cm^−2^) and also minimized the risk of interference by ascorbic acid (AA), uric acid (UA) and acetaminophen (AP), usually described as main the interferents in glucose assays. This fact is probably due to a direct electron communication bridged established by the carbon nanotubes between HRP and the electrode. The sensor also reveals a linear detection limit range from 0.50 µM to 1.00 mM for hydrogen peroxidase concentration, and a low detection limit of 0.50 ± 0.14 µM. In terms of stability, the biosensor retains 82% and 67% of the initial sensitivity, respectively, after 1 and 2 weeks of continuous measurements [51].

Electrode surfaces modified with self-assembled monolayers (SAMs) of thiols provide a simple route to tailored materials that can be further functionalized with AuNPs and enzymes. Gold(Au)NPs is another material applied in biosensor constructions. For example, several studies have reported that electrodes modified with Au-NPs enable the direct electron transfer from cytochrome *c* [73], myoglobin [74], HRP [75], superoxide dismutase [76] and bilirubin oxidase [77]. More recently, a study demonstrated that these modified electrodes could also allow a rapid direct electron transfer between laccase and Au-NPs modified electrodes, enabling a more efficient bioelectrocatalytic oxygen reduction [78]. Regarding the laccase biosensor, Lanzellotto and co-workers developed a nanostructure laccase-based electrochemical biosensor that exploits the beneficial features of functionalized fullerenols and AuNPs, such as high surface areas, conductivity, flexibility and reactivity. The results show that the assembled nanocomposite material (Au-AuNPs-fullerenols) reveals an enhancement of the electrochemical properties, and when immobilized with TvL presents a good stability, a reduction to 87% of the initial value of sensitivity after 120 days from preparation, a good reproducibility and an enhancement of the bioelectrochemical performance with a lower detection limit of 1.1 mg L^−1^∙mg∙L^−1^ [70].

Another device that has generated a growing scientific interest is the plasmonic nanostructure. As verified by Salamon and co-workers, this structure could be used as a solid-support planar proteolipidic membrane, that enables the study of the biochemistry and biophysics of membrane-associated receptors and enzymes using surface plasmon resonance (SPR) spectroscopy [79]. This structure has also been used for the study of pharmaceutical protein-protein interactions [80]. Therefore, the creation of a hybrid system that combines plasmonic nanoparticles with enzymes and at the same time links the biological and electronic functions is clearly advantageous. Several studies have verified that the previously described combination enhances the performance features of the biosensors, namely their sensitivity, selectivity, and limit of detection [81,82]. An example of this approach is the work performed by Ren and coworkers with enhancement of the electrocatalytic response of the sensor from 0.531 µA to 31.17 µA [83]. Also, Abel and collaborators in their study demonstrated that silver island films (SIFs), the type of plasmonic nanoparticle, can be incorporated into current enzyme-based biosensing applications for the detection of biotinylated compounds, revealing a increasing biosensor response [84].

## 4. Biosensosr Based on Transduction

### 4.1. Electrochemical Biosensors

Electrochemical biosensors are the oldest and more applied type, representing more than half of the biosensors reported in the literature [15,16,17]. They provide quantitative and semi-quantitative measurements through the use of an electrochemical transducer [14]. Further classified into amperometric, potentiometric, impedance, and conductometric sensors, these devices present a simple and quick methodology, high sensitivity, low-detection limits and low interferences from the matrix [14,16,17,63]. For all that, electrochemical detection appears to be a suitable option for the construction of miniaturized and portable biosensors [63,64]. However, some challenges persist, including high performance and cost effectiveness [16], so most recently published research is focused on the optimization of the biosensor structure [17]. Improvements in performance and stability have included new methodologies for biomolecule immobilization and the application of nanomaterials in biosensor construction [31].

#### 4.1.1. Amperometric Biosensors

The amperometric transducer is the most commonly used among electrochemical biosensor groups [14,18]. Amperometric biosensors are based on the measurement of the current resulting from the electrochemical oxidation or reduction of an electroactive species at a fixed potential, revealing a high sensitivity and a wide linear range that can be applied when drugs susceptible to oxidation are the subject of evaluation [14,15,18,85]. The majority of the available amperometric biosensors apply enzymes as biological recognition element [18,86]. The combination of the high specificity of the enzyme for recognizing the analyte with the direct amperometric transduction allows for a fast, simple and direct determination of a wide range of metabolites and therapeutic drugs [18]. However, there are still some challenges to overcome related to enzyme-based amperometric biosensors, including their reduced stability and electrochemical interferences present in complex samples matrices [14,87]. In contrast to enzyme-based biosensors, amperometric immunosensors work based in the detection of an electrochemical signal, generated from the binding of the antigen and labed antigen to the antibody immobilized electrode, that will be amplified by the labeled enzyme in the presence of the enzyme substrate, and thus lead to a specific analyte concentration [88]. Several amperometric immunosensors for clinical applications have been described [89,90,91]. However, reductants presents in biological liquids could interfere in the signal since they can be also oxidized, so to avoid the high noise signal present in these cases, mediators have been developed to shuttle the electrons between the enzyme and the electrode. For all that, the labeling amperometric immunosensor display some disadvantages such as the loss of the mediators, time-consumption and the need for more reaction steps. Zhang and co-workers developed a label-free amperometric biosensor for the detection of methamphetamine (MA). In this work, the biosensor was based on Prussian blue (PB), which works as a mediator and as an artificial peroxidase. The construct revealed a high selectivity, stability, sensitivity and also a decrease in the analytical time reaction with a linear range of 1.0 × 10^−8^ to 5.0 × 10^−6^ mol∙L^−1^ of MA, and a detection limit over 7.5 × 10^−9^ mol∙L^−1^ [88].

Grennan and collaborators described a device based on modified carbon paste screen-printed electrodes which enables direct coupling to take place between the redox centers of antigen-labelled HRP and the electrode surface. This novel method enables multi-competition analysis of analyte, in real time, on a single electrode, presenting a detection limit for atrazine of 0.1 ppb (0.1 µg∙L^−1^) [92]. Recently, Kim and co-workers also developed an immunosensor based on a cadmium sulﬁde nanoparticles modiﬁed-dendrimer bonded conducting polymer for the detection of chloramphenicol. The immunosensor exhibited a linear range of 50–950 pg/mL and a limit of detection of 45 pg/mL and was based on the competitive immune interaction between the free and labeled chloramphenicol for active sites of the Ab [93,94].

In the research on anti-inflammatory and analgesic drugs, amperometric biosensors are used extensively. For instance, when salicylate is the target, biosensors based on salicylate hydroxylase (SH), alone [95] or coupled to other enzymes like glucose oxidase, tyrosinase, hexokinase were proposed [96]. As a simple system, based on the SH electropolymerization onto a glassy carbon-working electrode with polypyrrole and glutaraldehyde, salicylates could be detect with a linear range between 2.3 × 10^−6^ and 1.4 × 10^−5^ mol∙L^−1^, where the blood serum samples showed a good correlation comparatively with the spectrophotometric method (Trinder) used as reference [95]. However, when a bi or tri immobilization was employed, a detection limit of 3.5 × 10^−6^ M was obtained, demonstrating that co-immobilization procedures can improve the sensitivity at lower working potentials [96,97].

Sanghavi and co-workers developed a biosensor based on a modified carbon nanotube paste electrode for individual and simultaneously determination of acetaminophen (ACOP), aspirin (ASA) and caffeine (CF). These led to a biosensor with detection limits of 2.58 × 10^−8^, 8.47 × 10^−8^ and 8.83 × 10^−8^ M for ACOP, ASA and CF, respectively [98]. Also, for the evaluation of ACOP, another amperometric biosensor was developed to detect its presence in three different pharmaceutical compositions. This biosensor was based on HRP and revealed a sensitivity of 74.9 mA∙M^−1^∙cm^−2^, a detection limit of 3.1 × 10^−6^ M and a linear range from 1.0 × 10^−5^ to 4.9 × 10^−4^ M APAP [99]. With the same propose, Messina and collaborators developed an on-line microfluidic sensing device with a linear range for the detection of paracetamol from 0.35 × 10^−6^–100 × 10^−6^ M and a detection limit of 3.0 × 10^−7^ M [100]. These recent biosensor models show good detection limit and in the case of the biosensor developed by Messina and collaborators, demonstrate the possibility of on-line detection of anti-inflammatory drugs without any compromise in the response.

Another relevant application of electrochemical sensors is new drug screening. This could be important in diseases that require the accurate selection of the most effective drug, like Alzheimer’s disease (AD). With the purpose of developing a simple drug sensitivity test, Du and collaborators developed a biosensor to investigate the efficiency of the inhibition of the activity of immobilized AChE by neostigmine and galantamine (drugs associated with AD). The assembled construction reveals an amplification of the sensitivity, with better accuracy with other conventional assays [101]. A reliable example of the application of these biosensors is based on cytochrome P450 family enzymes (CYP 450). The CYP 450 family consists on a group of heme-containing enzymes implicated in the metabolism of xenobiotics in the body, which makes them a target in pharmaceutical research for the screening of lead compounds in drug development [102]. These systems are characterized by electron transfer during substrate conversion and the electrochemistry mechanism underlying the research using several metal electrodes (e.g., Au, Pt and Tin oxide) and non-metal electrodes (e.g., glassy carbon, pyrolytic graphite, edge-plane graphite and carbon cloth) [103]. In recent years, several CYP enzymes such as CYP2B4, CYP1A2 and CYP3A4 have been immobilized for the detection of multiple compounds including aminopyrine, benzphetamine, phenobarbital, clozapine, quinidine, nifedipine, alosetron, and andondansetron [102]. Panicco and coworkers described a micro-machined eight-electrode array for the amperometric measure of K_M_ and k_cat_ values of these phase-1 drug metabolizing polymorphic enzymes. In this study, human P450 2D6 and 2C9 were engineered and covalently linked to a surface of a modified gold electrode and the results were validated using warfarin and bufuralol, which are marker drugs for the 2C9 and 2D6 enzymes, respectively [102,104]. Recently, based on CYP 450, Baj-Rossi and collaborators described the characterization of an electrochemical biosensor in order to monitor naproxen. The dynamic linear range for the amperometric detection of naproxen had a major and minor detection concentration, respectively, of 300 µM and 16 ± 1 µM, included in the physiological range (9–300 µM). This system allows real time naproxen profiling and represents an innovative point-of-care solution [105].

#### 4.1.2. Potentiometric Biosensors

Potentiometric biosensors are based on the use of an ion-selective electrode and ion-sensitive field effect transistor for obtaining the analytical information. In these sensors, the signal resulting from a biorecognition process is converted into a potential measurement to provide an analytical signal [14].

In addition to the amperometric ones, potentiometric transducers are often used in electrochemical biosensors due to their long durability and mechanical stability. Further advantages come from their simple instrumentation, low cost, and their ability for continuous monitoring [17]. The lack of sensitivity between concentrations within the same order of magnitude and high occurrence of non-specific binding effects when compared to amperometric counterparts are some of the major drawbacks [14,63]. Another relevant problem with this device is the leaching of the membrane components into samples. This can be solved by reducing the electroactive components in polymeric membranes [17].

Potentiometric biosensors have been used for the detection of compounds of pharmaceutical interest for a long time. Ameer and colleagues developed a potentiometric biosensor based on entrapment of a tyrosinase into a conducting polypyrrole film on a platinum electrode for the detection of catechols. This device presented a sensitivity of 10 mV/µM with a response time of 80 s and the lowest detectable concentration was 1.0 µM [106]. More recently, the same research group built and compared two electrochemical sensors, a potentiometric and an amperometric device, also based into enzyme immobilization via entrapment into polypyrrole films. The co-immobilization of purine nucleoside phosphorylase and xanthine oxidase enabled the detection of phosphates with a lower linear concentration range and minimum detectable concentration level (20–200 µM, 20 µM) when compared to amperometrics (0.1–1 mM) [107]. For the determination of epinephrine, Mataveli and coworkers proposed a potentiometric biosensor based on the packaging of a modified carbon paste containing a polyphenol oxidase extract into a polyethylene tube. This miniaturized biosensor provide a linear range from 8.00 × 10^−9^ to 8.00 × 10^−4^ mol∙L^−1^ for medicine samples and from 8.00 × 10^−7^ to 8.00 × 10^−3^ mol∙L^−1^ for blood samples [108].

Recently, a new approach was developed for the screening and characterization of natural compounds that may act as potential competitive reversible inhibitors of acetylcholinesterase (Ach). Natural compounds may constitute interesting therapeutic drugs for the treatment of brain diseases as Ach inhibitors, due to its role in the termination of impulse transmission in the cholinergic synapses through the hydrolysis of acetylcholine. Consequently, the developed method was applied to the Ach inhibitor galantamine using an acetylcholine-selective electrode, exhibiting a linear range from 2 × 10^−8^ to 1 × 10^−6^ M, a limit of detection of 5.4 × 10^−9^ and inhibition constant K_I_^Gal^ of 2.0 × 10^−7^ ± 0.1 × 10^−7^ M [109].

### 4.2. Optical Biosensors

Over the last decades, fast and significant advances in instrumentation and experimental design have increased the applicability of optical biosensors in many fields, including drug discovery [13,14]. Optical biosensors are constituted by a light source that generates light with specific characteristics, a modulating agent, a sensing area and a photoreceptor [16]. In general, these sensors can measure changes in local refractive index at the sensor surface but, specifically, they can exploit surface plasmon resonance, waveguides and resonant mirrors to analyse biomolecular interactions. However, most of these biosensors exploit the evanescent waves phenomenon, an electromagnetic field created by the total internal reflection of light at a solution-surface interface, to characterize the interaction between enzymes/Ab and analyte. Thus, optical sensors enable the determination of the affinity and kinetics of a wide variety of molecular interactions in real time, with or without the need for a molecular tag or label [13].

The amount of information provided by these devices is large, while binding rates and binding levels can be interpreted in order to obtain the specificity, kinetics and affinity of the interaction and/or analyte concentration. Other advantages are the low signal-to-noise ratios, the low reagent volumes required for analysis and, particularly, the possibility of performing real-time analysis [16]. The direct optical biosensors, as the name suggests, allow effective real-time analysis without the need of labelling the analyte or the enzyme/Ab of complex matrices, avoiding the separation step to remove free from bound label [16,18]. However, some optical devices, the indirect biosensors, incorporate suitable labels including fluorophores to carry out the analysis [16].

#### 4.2.1. Surface Plasmon Resonance Based Biosensors

Surface Plasmon Resonance (SPR), is an optical phenomenon, applied in biosensor technology that occurs as a result of the total internal reflection of monochromatic and polarized light [15]. These devices measure in real-time the light absorbed or emitted, as a result of a biological and/or chemical reaction, which are related to the quantity of complex formed between the immobilized molecule on the sensor surface and a molecule in solution without the need of any labeling [12,14,15,110,111]. Through the knowledge of the protein concentration, the percentage of compound bound and half-life of the drug can be deduced [13]. As a device that could be applied directly to monitor the binding of relatively low molecular weight compounds to immobilized drug targets, with the capacity to provide a kinetic data for drug-target (e.g., enzymes, Ab, receptors) interaction, these sensors are extremely advantageous in identification, screening and optimization of leading molecules for drug discovery and ADME analysis [112].

In the case of imunosensors, the stable Ab immobilization via a biotin-avidin interaction or covalently via amine, thiol or aldehyde chemistry allows the biosensor regeneration, increasing its lifespan (50–100 analytical cycles) [18]. In particular, the Ab immobilization via biotin-avidin appears to avoid non-specific avidin adsorptions, increase the storage time of biosensor and enables its miniaturization [63]. However, non-specific interactions with the biorecognition elements can be verified, which suggests a lack of specificity when compared with indirect optical methods [18]. Another concern is its inability to analyze low molecular weight compounds (<500 Da) because, as SPR detects the mass of the analyte, smaller molecules prevent an adequate measurement [15]. Nevertheless, improvements in signal-to-noise ratios increased the chances of detection of very small analytes [14]. Indeed, Kim and coworkers have described a portable device designed for the detection of low molecular weight analytes [63].

Several publications have appeared in the last decade describing pharmacological applications of SPR methodology. The SPR technology has been increasingly used in the construction and development of new biosensors for the assessment of kinetic parameters, including in the recognition and stability in a couple of drugs. Many efforts has been made in order to improve the SPR low molecular weight drug detection capacity by increasing sensitivity through improvements in hardware, software and data evaluation [110]. Based on that, recently we saw the use of such technology as a toll in fragment-based drug design [82]. Milkani and coworkers developed a surface plasmon resonance sensor capable of directly detecting AChE inhibitors. As referred above, AChE plays an important role in Alzheimer’s disease, because its “reversible” inhibition has a great therapeutic potential. In this work, the authors used neostigmine and eserine (compounds used in the treatment of the disease) as inhibitors, and evaluated their binding affinity (K_A_) to AChE by observing the changes in the refractive index values. The results showed that neostigmine presents a higher affinity (3.8 × 10^−3^ M^−1^) than eserine (1.7 × 10^−3^ M^−1^) for AChE. Also, the results indicated that the modification in the refractive index compared with the relatively small molecular weight inhibitors, must be due to a shift in enzyme conformation as a result of inhibitor binding in the active site [113].

Markgreen and collaborators applied this technology in order to establish a relationship between structure and interaction kinetics for HIV-1 protease (retroviral aspartyl protease essential for the life-cycle of HIV). For traditional assays HIV-1 protease inhibitors are a challenge, since this enzyme is very instable and it is thus difficult to determine the inhibitory constants of the most potent inhibitors. However, with SPR technology the association (K_on_) and dissociation (K_off_) rates for the interaction of enzyme-inhibitor can be assessed, and so the equilibrium dissociation constant (K_p_ = K_off_ ÷ K_on_) can be calculated from independent variables. This is corroborated by the results shown for saquinavir. As the inhibitor who presents the highest affinity, with a K_D_ of 0.315 ± 7.42 × 10^−2^ nM, it also reveals the lowest dissociation rate constant of all inhibitors analyzed (K_off_ 2.27 × 10^−4^ ± 3.04 × 10^−5^, K_on_ 8.17 × 10^5^ ± 1.61 × 10^5^ M^−1^∙s^−1^), which confirms that association and dissociation rates constitute an important parameter of drug-target interaction showing that the optimization and screening of inhibitor efficacy and potency could be achieved with the information of both high association and low dissociation rates rather than by high affinity alone [114].

Another example of the application of this technology are the studies involving the kinases family. As a molecule that catalyzes the transfer the γ-phosphate group of ATP or GTP to the hydroxyl group of serine, threonine, or tyrosine on a substrate protein (often as a response to membrane receptor activation), constitutes an important target in drug evaluation once the phosphorylation status of the protein is crucial in the cellular signaling cascades. Malfunctions in the process can have pathological consequences, such as cancer and inflammatory disease, highlighting the importance of the studies of kinases [115]. Nordin and co-workers performed a study where the kinetic profiles of small molecules with protein kinases were evaluated, revealing once again that the use of kinetic data directly links a modification in structure to changes in recognition (*k*_a_), stability (*k*_d_), or both, which shows that the affinity differences between the binders could be related either to each rate constant alone or to both rate constants concurrently [58].

Recently, Das and collaborators constructed a prototype nanoparticle sensor based on Localized Surface Plasmon Resonance (LSRP) to detect drug binding on human membrane cytochrome P4503A4 (CYP3A4). As it is known, CYP3A4 is one of the most important enzymes in the metabolism of xenobiotic drugs. However, it presents a propensity to aggregate and so it becomes difficult to study the drug binding in solution and on a specific surface, so the authors developed a nanodisk to functionalize and stabilize the monomeric CYP3A4 on Ag nanoparticles. The result was the detection of different types of drugs by shifting. As LSR is similar to SPR, the measurement is based on the changes in the refractive index, so when the analyte binds to the device, the maximum extinction wavelength (λ_max_) of the nanoparticle shifts [116].

Recently, biosensor immunoassays were developed for monitoring chloramphenicol (CAP) residues. This study consisted primarily on the screening of anti-CAP Abs to improve biosensor specificity and reduce the detection limits using an assay based on the inhibition of the binding of two different polyclonal anti-CAP Abs to immobilized CAP on a sensor chip with two different immobilization strategies: CAP base surface and CAP surface. The best assay was obtained with Ab 1 on the CAP base surface due to its very low detection limit (0.1 µg∙L^–1^) and the decreased consumption of Ab, four times less. Then, five different phenicols (chloramphenicol base, chloramphenicol succinate, thiamphenicol, florphenicol, chloramphenicol glucuronide) and other commonly used antibiotics were tested in order to determine the specificity of the assay [117].

#### 4.2.2. Flourescence Based Immunosensors

Fluorescence protein-based biosensors are highly sensitive tools that allow resolving molecular events occurring in only hundreds of milliseconds, demonstrating that information can be transmitted through temporal patterns in signaling pathways [118]. In particular, drug discovery is a particular area that may benefit from the application of such biosensors. This technique may deepen the knowledge of drug-target biomolecule interactions through the study of the mechanisms underlying ligand bias and the ability of ligands to selectively stabilize receptor conformations that stimulate or inhibit subsets of receptor activities [118].

Fluorescence biosensors are constituted by biological or biomimetic recognition elements, where one or several fluorescent coupled probes (enzymatically, chemically or genetically), are capable of recognizing a specific analyte and the signal generated by this recognition process is transduced into a fluorescent signal which can be readily detected and measured [119]. However, the majority of analytes and biological sensing elements lack intrinsic fluorescence properties. In this case, the interaction of this elements is transduced to an optical signal by coupling fluorescent molecules to elements involved in recognition event [14]. The chemical nature and the physicochemical properties fluorescent transducer must be taken into account in the design of biosensor in order to obtain an optimal signal. Some signals may change depending on the environment conditions, including pH, polarity and electrochemical composition. Since the biorecognition event lead to conformation rearrangements that produce changes in environment, the fluorophore emits a signal when it sense its perturbation or may be quenched when the label is in the proximity of a ligand or another protein. Moreover, the selection of the position to introduce the fluorescent label into the biological recognition element is also crucial and should result in a low perturbation of the stability and the recognition event and high sensitivity detection. In general, the fluorescent attachment is based in attachment of chemical groups and/or in the genetic fusion of green fluorescent proteins (GFP) or derived proteins. However, the application of labels through chemical methods have the advantage of introducing the label at any position [120]. Regarding the application of enzymes as recognition element, as the catalysis is not desirable in some cases, some enzymes have been modified to impair their activity and conserve only the ligand binding property [120]. Also, Ab-based fluorescent biosensors are beginning to receive attention as suitable biomolecules for diagnostics in homogeneous immunoassays or as imaging probes [121].

Fluorescent biosensors thus provide a sensitive means of probing ions, metabolites, protein biomarkers and other molecules which can be used as therapeutic compounds. Additionally, they can provide information about the activity or conformational status of a specific analyte in complex matrices such as serum and cell extracts, allowing the monitoring of dynamic processes in a real-time mode and the study the behavior of enzymes in terms of function and regulation [119]. The major drawback of fluorescence technology are the additional complexity of instrumentation, in either the time or frequency domains [14].

Calmoduilin (CaM) is a calcium binding proteins that represents an important drug target for many drugs including antipsychotic, smooth muscle relaxants, antitumoral and α-adrenergic blocking agents, among others [120]. Therefore, CaM has been used as biological recognition element for the design of fluorescent biosensors in order to study interactions between this and other molecules, for example, the interaction between the CaM coupled to three different fluorophores and phenothiazines and related tryclic antidepressants [120]. Recently, a new fluorescent biosensor based on a human CaM (hCaM) was constructed for the detection of calcium ion (Ca^2+^) and CaM inhibitors in solution. The bionsensor was applied to analyze the effect of Ca^2+^ on the binding of trifluoroperazine to CaM, verifying that K_D_ ranged from 5.7 ± 0.5 and 0.5 ± 0.07 µM and depends on Ca^2+^ levels (0–10 µM). Additionally, this sensor appears to be suitable for discovering xanthones with anti-CaM properties from the fungus *Emericella*, displaying excellent affinities with K_D_ ranging from 4 to 498 nM [122].

Cyclin-dependant kinases (CDK/cyclins) are heterodimeric protein kinases that play a central role in coordinating cell growth and division. Due to their role in biological systems, CDK/cyclin levels and activities are frequently altered in human cancers, contributing to sustain aberrant proliferation in cancer cells. So, CDK/cyclins constitute attractive pharmacological targets for the development of anti-cancer therapies. CDKACT are activity-based biosensors constituted of a peptide substrate where a fluorescent label is coupled to the phosphorylation site and a phosphoaminoacid domain that recognizes the phosphorylated peptide sequence. During phosphorylation by active CDK/cyclin complexes, CDKACT detects changes in fluorescence intensity, related with kinase activity. Then, it allows to monitoring CDK/cyclin activity in real-time, detecting differences in CDK/cyclin activities in response to drugs [119]. With respect to immunosensors approaches, Muriano and coworkers constructed a fluorescent device based on immobilization of purified IgGs (Ab-143 and Ab-pre) for the detection of methylboldenone (MB), an androgenic anabolic steroid. The design of this immunosensor is based on two-photon fluorescence spectroscopy coupled to a resonant double grating waveguide structure and specific anti-MB abs are immobilized onto a resonant Ta_2_O_5_ sensing chip activated with phosphonohexanoic acid spacers. The detection relies on a direct competition using a boldenone-rhodamine conjugate as fluorescent competitor, enabling a synthetic steroid detection down to 0.1 µg∙L^−1^ [123].

## 5. Conclusions and Future Perspectives

Since their first appearance, biosensors have become a widespread technology. First applied in routine analysis and diagnosis, they suddenly revealed their potential to enable screening any receptor-ligand complex. Also, with the advances in immobilization strategies, alongside with the advent of nanomaterials, biosensors will become a more robust and reliable technology. Succinctly and until the present, continuous measurement biosensors for the monitor of substances like glucose and other endogenous compounds have already been reported. However, few examples are described for this application in continuous monitoring of pharmaceutical compounds, with only one enzyme-based biosensor reported. The improvements achieved in sensitivity of biosensors through the use of redox cycling, are extremely relevant for drug screening analysis, ensuring a better amplification and enabling the creation of a next generation of ultrasensitive biosensors. Also, the application of SPR technology to biosensors will allow the assessment of kinetic data from compounds binding to specific therapeutic targets, yielding new information for the optimization of pharmacological screening. Due to its capacity to measure increasingly lower molecular weights, SPR is starting to appear as a tool for FBDD, leaving behind the high throughput screening mode. Finally, regarding kinetic studies of ADME profiles, up until now few studies were described in the literature. Therefore, further investigation in this domain should be performed in order to establish the biosensors as a screen that could be applied in all the ADME stages.

In conclusion, despite the difficulties in achieving reproducibility, stability and sensitivity of these types of sensors, all of them have proved to be a useful tools for the pharmaceutical industry in areas that encompass almost all stages of the drug discovery process. Thus, it is expected in the next years, the availability of an increasing number of devices, upgraded by simple and low cost methods, will allow the discover of new therapeutic agents.
